# Exploring the Relationship Between Gut Microbiota and Sarcopenia Based on Gut–Muscle Axis

**DOI:** 10.1002/fsn3.4550

**Published:** 2024-10-21

**Authors:** Wei Li, Ren‐Wang Sheng, Mu‐Min Cao, Yun‐Feng Rui

**Affiliations:** ^1^ Department of Spinal Surgery Unit 1 Hanzhong Central Hospital of Shaanxi Province Hanzhong Shaanxi China; ^2^ Department of Orthopaedics Tianjin Hospital of Ningqiang Hanzhong Shaanxi China; ^3^ Department of Orthopaedics, School of Medicine, Zhongda Hospital Southeast University Nanjing Jiangsu China; ^4^ School of Medicine Southeast University Nanjing Jiangsu China; ^5^ Multidisciplinary Team (MDT) for Geriatric Hip Fracture Management, School of Medicine, Zhongda Hospital Southeast University Nanjing Jiangsu China; ^6^ Orthopaedic Trauma Institute (OTI) Southeast University Nanjing Jiangsu China

**Keywords:** gut, gut microbiota, gut–muscle axis, muscle, sarcopenia

## Abstract

Sarcopenia, as a disease characterized by progressive decline of quality, strength, and function of muscles, has posed an increasingly significant threat to the health of middle‐aged and elderly individuals in recent years. With the continuous deepening of studies, the concept of gut–muscle axis has attracted widespread attention worldwide, and the occurrence and development of sarcopenia are believed to be closely related to the composition and functional alterations of gut microbiota. In this review, combined with existing literatures and clinical reports, we have summarized the role and impacts of gut microbiota on the muscle, the relevance between gut microbiota and sarcopenia, potential mechanisms of gut microbiota in the modulation of sarcopenia, potential methods to alleviate sarcopenia by modulating gut microbiota, and relevant advances and perspectives, thus contributing to adding more novel knowledge to this research direction and providing certain reference for future related studies.

## Graphical abstract

This review summarizes the role and impacts of gut microbiota on the muscle, the relevance between gut microbiota and sarcopenia, potential mechanisms of gut microbiota in the modulation of sarcopenia, potential methods to alleviate sarcopenia by modulating gut microbiota, and relevant advances and perspectives, thus contributing to adding more novel knowledge to this research direction and providing certain reference for future related studies.

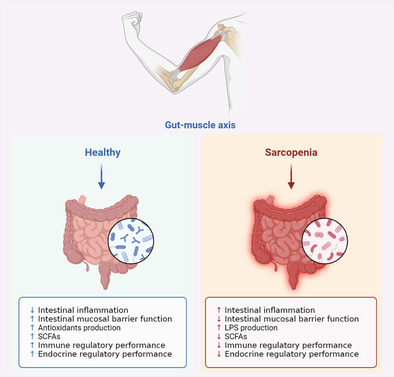

## 1 Introduction

Recently, the social problems brought about by the aging of global population have become increasingly prominent, and advocating the concept of healthy aging is particularly significant (Clynes et al. 2021; Kirk et al. 2024; Zuo, Li, et al. 2023). Sarcopenia is characterized by a progressive decline in skeletal muscle mass, strength, and function with age, which is mainly divided into age‐related sarcopenia (also known as primary sarcopenia) and disease‐related sarcopenia (also known as secondary sarcopenia) (Gielen et al. 2023; Xie et al. 2021). According to the statistics of European Working Group on Sarcopenia, the global prevalence of age‐related sarcopenia ranges from 10% to 27%, with an average prevalence of 17.7% (Cruz‐Jentoft et al. 2019; Sayer and Cruz‐Jentoft 2022). Current treatments for sarcopenia are limited to exercise and nutrition, with limited efficacy (Dent et al. 2018). Thus, it is necessary to actively explore new means to improve and enhance the muscle mass and function of the elderly, thus safeguarding the ability of the elderly to live independently in daily life, which is also in line with the vision of global healthy aging (Zhang, Lu, et al. 2021).

The human gut harbors a microbial community of 10–100 trillion microbes, and the genes carried by these microorganisms participate in the modulation of various physiological functions in human body, such as immune function, brain activity, and intestinal barrier integrity (Zhang, Song, et al. 2024; Zhang, Wu, et al. 2024). Recent studies have indicated that gut microbiota can influence the metabolism, quality, and function of skeletal muscle by altering host glucose and lipid metabolism, amino acid metabolism, insulin sensitivity, inflammatory response, stem cell homeostasis, and so on (Zhang, Cheng, and Hu 2022; Zhang, Cao, Li, Chen, et al. 2022; Zuo, Pang, et al. 2023). Moreover, the recently proposed concept of gut–muscle axis has gradually attracted widespread attention worldwide, and the occurrence and development of sarcopenia are regarded to be closely associated with the species composition and functional alterations of gut microbiota (Feng et al. 2020; Zhang, Cao, Li, Lu, et al. 2022). In addition, previous studies have gradually focused on the changes of gut microbiota in the patients with sarcopenia. Fielding et al. (2019) investigated that *Prevotellaceae* and *Barnella* might be involved in maintaining muscle strength in the elderly population. A study on the correlation between dietary habits, microbial components, and personal health among 178 elderly participants suggested that both community‐based and long‐term hospitalized elderly participants had unique microbial components associated with aging (Davinelli and Scapagnini 2022). Ren et al. (2021) also suggested that the diversity of gut microbiota in patients with sarcopenia and cirrhosis significantly decreased. Margiotta et al. (2021) investigated that compared to the patients without sarcopenia, elderly patients with chronic kidney disease and sarcopenia exhibited significant alterations in the microbial components, including a significant enhancement in the abundance of *Akkermania mucinophila*. Furthermore, therapeutic strategies for modulating gut microbiota, such as probiotics and prebiotics, exercise, dietary intake, fecal microbiota transplantation (FMT), and so on, have showed huge potentials in reducing muscle loss (Song et al. 2022; Zhang, Cao, Li, Zhang, et al. 2022; Zhou et al. 2023). Regarding this, this review mainly focuses on the effects of gut microbiota on the muscle, relevance between gut microbiota and sarcopenia, potential mechanisms of gut microbiota in the modulation of sarcopenia, and potential methods to alleviate sarcopenia by modulating the gut microbiota, thereby providing reference for the future researches and development of intervention methods.

## 2 Role and Impacts of Gut Microbiota on the Muscle

Muscles account for 40% of the total body mass and play a crucial role in regulating body temperature and maintaining glucose and amino acid homeostasis (Coletta and Phillips 2023; Zanker et al. 2023). Due to the secretion characteristics of muscles, the decline of muscle mass and function may cause metabolic disorders and communication barriers between organs in human body (Jiang et al. 2023). Moreover, sarcopenia is common in the elderly, as well as in patients with acute and chronic muscle wasting diseases, and is closely related to the prognosis of these patients, and the role and impacts of gut microbiota on muscles are mainly reflected in the following aspects, including muscle mass, muscle composition, and muscle function.

### 2.1 Muscle Mass

Aging process is often accompanied by chronic low‐level inflammatory reactions, and the enhancement of endotoxin levels caused by the alterations in gut microbiota is one of the main causes of age‐related inflammation (Zhang, Cao, Li, Dai, et al. 2023). Siddharth et al. (2017) compared the rats with age‐related sarcopenia with those with normal muscle mass and found that the microbial components were significantly different, and the muscle cell group also exhibited different functions, such as reducing the expression of genes involved in the biosynthesis of carbohydrate, protein, lipid digestion, and vitamin, thus resulting in a vital decrease in the bioavailability of nutrients. Other studies have shown that butyrate (a short‐chain fatty acid produced by gut microbiota that inhibits histone deacetylase and has anti‐inflammatory and prosynthetic metabolic effects) is related to physiological age‐related muscle mass loss, and the supplementation of butyrate can enhance muscle mass and cross‐sectional area in the elderly mice (Picca et al. 2022; Zhang, Cao, Li, Zhang, et al. 2022). These studies emphasize the role of gut microbiota‐derived metabolites in promoting the muscle synthesis, including not only short‐chain fatty acids represented by the butyrate but also several phenolic compounds (Lv et al. 2021). However, there is still a lack of direct evidence to prove a close link between the composition of human gut microbiota and muscle mass, especially without an association with age‐related sarcopenia (Chen et al. 2022; Grosicki, Fielding, and Lustgarten 2018).

### 2.2 Muscle Composition

Aging causes the transformation of muscle fiber types from fast muscle fibers to slow muscle fibers, and the composition of myosin heavy chain isoforms also changes, thereby reducing the overall muscle strength and enhancing the risk of falls (Okamura et al. 2022; Zhang, Cao, Li, Dai, et al. 2022). However, researches have not yet elucidated the mechanism behind this phenomenon, and the alterations in the gut microbiota of the elderly may be involved in this process. Bäckhed et al. (2007) indicated that even when germ‐free mice were fed high‐sugar/high‐fat foods, the mice continued to exhibit a lean phenotype. Yan et al. (2016) suggested that when the fecal microbiota of obese mammals was transplanted into the gut of GF mice through the means of FMT, GF mice replicated the muscle fiber characteristics of the donor, manifested as a higher proportion of slow‐contracting fiber and a decreased proportion of fast IIb fiber. In addition, muscles are also one of the significant metabolic organs in the human body, mainly using glucose and lipids to generate energy (Frampton et al. 2020). Gut microbiota involved in energy metabolism regulates intramuscular fat and other components, and changes in the gut microbiota can also result in fat to infiltrate the bone, ultimately leading to a triple imbalance of muscle, bone, and adipose tissue, which is also known by a unique term osteosarcopenic obesity (OSO) (Pedraza‐Vázquez et al. 2023; Perna and Rondanelli 2023).

### 2.3 Muscle Function

The sum of age‐related changes in muscle size and composition leads to a decline of muscle function, ultimately influencing the physical performance and the ability to live independently (Lima et al. 2023). The weakness and mobility caused by aging are related to varying degrees of dysbiosis of the gut microbiota (Lahiri et al. 2019; Zhang, Li, et al. 2021). Specifically, dysbiosis is characterized by an increased proportion of opportunistic microorganisms (members of enterobacteria and *Clostridium* spp.), decreased SCFAs, and increased metabolites such as trimethylamine (TMA), secondary bile acids, and p‐Cresol. Van Tongeren et al. were among the first to correlate the changes of gut microbiota with physical function. After stratifying subjects using frailty scores, they observed that *Lactobacillus* was significantly reduced in frailty elderly individuals (reduced to 1/10 of the control group), and the number of *Enterobacteriaceae bacteria* enhanced accordingly (approximately six times) (Calon et al. 2019; van Tongeren et al. 2005). To explore the link between gut microbiota and physical function in elderly people more intuitively, a team from Nutrition Research Center on Aging at Tufts University in the United States conducted a simplified physical fitness assessment on a total of 29 people, including standing balance, 400 m walking, and chair‐standing tests. Based on the scores, the research subjects were grouped into low or high functional groups, and human body composition measurements and fecal analysis were also performed (Fielding et al. 2019). The research results indicated that the elderly people with a higher body weight and better physical performance have a higher abundance of microbial communities, such as *Prevotella* and *Barnella*, and mice with high functional microbiota colonization have higher grip strength (Farini et al. 2023). These alterations have also been verified in the intervention studies with probiotics and prebiotics, where the supplementation with *Lactobacillus plantarum TWK10* could enhance the proportion of type I fibers in the gastrocnemius muscle of rodents and improve exercise endurance (Chen et al. 2016; Huang et al. 2015, 2019).

## 3 Relevance Between Gut Microbiota and Sarcopenia

**FIGURE 1:**
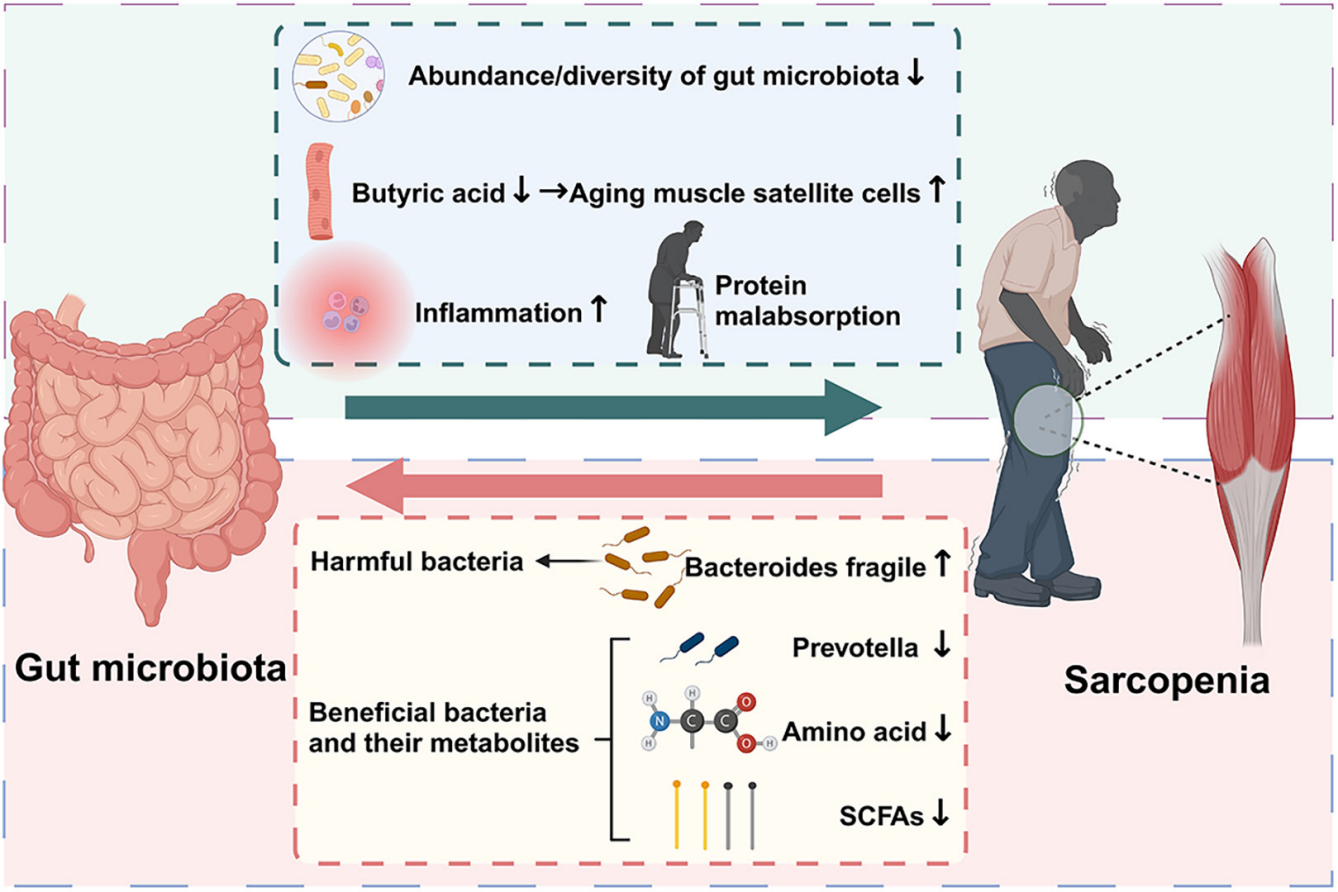
Relationship between gut microbiota and sarcopenia.

The health status of gut microbiota is closely related to sarcopenia, and the link and interaction between the two are complex and intricate (Rashidah et al. 2022) (Figure 1). On one hand, the diversity and composition of gut microbiota and its interactions with secondary metabolites are inextricably linked to sarcopenia (Giron et al. 2022; Wang et al. 2022). On the other hand, factors such as dietary structures and the living environment also play an undeniable role in it (Park et al. 2022). Previous study has revealed that the occurrence and development of sarcopenia are closely related to the changes of gut microbiota (Lee et al. 2022). Various sarcopenia‐related diseases, such as myasthenia gravis, inflammatory myopathy, glycogen storage disease, spinal cord injury, and cachexia, are also associated with reduced abundance and diversity of gut microbiota (Lee, Kim, et al. 2023; Yan et al. 2023; Yang et al. 2022). In addition, the progression of sarcopenia can affect the microbial components and the level of secondary metabolites, resulting in a chain reaction (Aliwa et al. 2023). For example, the accumulation of metabolites produced during the progression of sarcopenia can lead to excessive proliferation of pathogenic bacteria (represented by *Bacteroides fragile*) and affect the composition and content of healthy microorganisms and induce the dysbiosis of microbiota, thus leading to a decline in the level of short‐chain fatty acids, damaging the intestinal function, and affecting the muscle health (Baek et al. 2023; Han et al. 2023).

Gut–muscle axis plays a crucial role in modulating age‐related muscle health, and the gut microbiota can influence muscle mass and function through various factors such as inflammation, immunity, substance metabolism, endocrine function, and insulin sensitivity (Ticinesi et al. 2017; Ticinesi, Nouvenne, et al. 2019). In terms of population‐based studies, Zhao et al. (2023) observed some components of gut microbiota related to the characteristics of sarcopenia via Mendelian randomization, providing evidence for the strategies to prevent and treat sarcopenia by regulating gut microbiota, which contributes to better understand the gut–muscle axis. Kang et al. (2021) recruited 87 individuals, including 11 patients with sarcopenia, 16 patients with potential patients, and 60 healthy control subjects, and evaluated their collected feces. The results indicated that the diversity, structural composition, and functional changes of gut microbiota may lead to a decrease in skeletal muscle mass and function, providing novel directions for the diagnosis and treatment of sarcopenia. A study from Spain randomly divided 60 elderly individuals into an experimental group and a control group. The experimental group received prebiotics containing 3375 mg of inulin and 3488 mg of oligofructose daily, while the control group received the same dose of maltodextrin daily. After taking them separately for 13 weeks, the elderly in the experimental group felt significantly less fatigue per week than the control group, and their right‐hand grip strength was significantly stronger than the control group, proving the positive regulatory effects of gut microbiota on human skeletal muscle function (López‐Bueno et al. 2022). Barton et al. (2018) indicated that the differences in the composition of gut microbiota between professional rugby players and control group were matched with significant differences in fecal metabolomics. Compared with the control group, the genes involved in carbohydrate and amino acid metabolism in the feces of athletes are more representative, resulting in higher concentrations of acetic acid, butyric acid, and propionic acid in the feces. A recent review also summarized the changes of gut microbiota associated with frailty and sarcopenia, suggesting the possible role of chronic low‐grade inflammation in it, and inflammation characterized by elevated levels of blood inflammatory markers may result in the development of frailty and sarcopenia in older adults. The changes of gut microbiota and its related metabolites might be crucial regulatory factors mediating chronic inflammation in frailty and sarcopenia (Xu et al. 2021). Burtscher et al. (2022) also proposed that exercise can effectively combat age‐related muscle loss, and the type and intensity of exercise can trigger the alterations of the composition and function of gut microbiota, enhance the diversity of gut microbiota, and bring health benefits to the host. The presence of gut microbiota ensured the normal muscle adaptation to exercise, promoted dietary protein digestion and amino acid absorption, and reduced the muscle loss, and strategies to combat muscle loss should also consider the influences of exercise plans and dietary interventions (balanced dietary intake of protein and fiber) on gut microbiota (Dao et al. 2020; McCormick and Vasilaki 2018).

As one of the four major tissues in the human body, muscles play a crucial role in modulating body movement and glucose and lipid metabolism homeostasis (Strasser et al. 2021). As for the animal‐based studies, Chen et al. (2024) revealed through a study that gut microbiota and its related metabolites were involved in regulating homeostasis of muscle satellite cells during the process of aging, suggesting that the decrease in the levels of butyric acid of muscles may be one of the reasons for the loss and functional defects of aging muscle satellite cells, and providing novel targets and ideas for developing treatment strategies to prevent skeletal muscle aging and related diseases. Liu et al. (2023) revealed that a wide diurnal temperature range was positively correlated with the prevalence of sarcopenia in humans. This phenomenon also existed in mice and is related to the gut microbiota. Fluctuating temperature exposure (10°C–25°C) accelerated muscle atrophy in middle‐aged male mice, suppressed motor performance, and altered the composition of the mice microbiota, and fluctuating exposure to the gut microbiota of transplanted mice reproduced adverse effects on muscle function. As a degradation product of lysine, aminoadipic acid was reported to be a glutamine synthetase inhibitor and a potential regulator of glucose homeostasis. Mechanically, the altered gut microbiota enhanced circulating aminoadipic acid, which impacted the mitochondrial function by inhibiting mitochondrial autophagy in vitro, and the supplementation of *Eubacterium* can relieve the muscle atrophy and dysfunction caused by temperature fluctuations. Liu et al. (2023) analyzed fecal metagenomes and untargeted metabolomics of 141 cases of sarcopenia and 142 cases of non‐sarcopenia, suggested the key differential bacteria (*Prevotella*), explored the effects of *Prevotella* on muscle mass and muscle function in elderly mice, and indicated that *Prevotella* can delay age‐related muscle mass and function decline, which had high clinical translational application values and prospects. This study verified the role of *Prevotella* in sarcopenia through animal experiments, providing a novel idea and target for the future microbial interventions in sarcopenia and broadening a new therapeutic strategy for the clinical treatment of sarcopenia. Puerarin (daidzein 8‐C‐glucoside) is an isoflavone derivative isolated mainly from the roots of *Puerariae lobate* (Willd) Ohwi. As a natural active ingredient, puerarin has hypoglycemic, lipid‐regulating, anti‐inflammatory, antioxidant, neuroprotective, and vasodilator effects. It has been widely used as an adjunctive treatment for diabetes, coronary heart disease, retinal artery occlusion, and ischemic cerebrovascular disease. Moreover, Yang et al. (2022) also revealed that feeding puerarin can significantly improve grip strength and skeletal muscle (soleus and extensor digitorum) contraction strength in rats, and increase the proportion of type II muscle fibers in the skeletal muscle cross sections. These results were mainly obtained by enhancing the content of short‐chain fatty acid (SCFA)‐related bacteria (*Peptococcaceae* and *Closteriales* families) and downregulating the proportion of muscle atrophy‐related bacteria (*Prevotellaceae*/*Bacteroidaceae*). Through these changes in gut microbiome, puerarin increased the production of SCFAs including acetic acid, propionic acid, and n‐butyric acid and might increase ATP synthesis and the skeletal muscle strength.

## 4 Potential Mechanisms of Gut Microbiota in the Modulation of Sarcopenia

**FIGURE 2:**
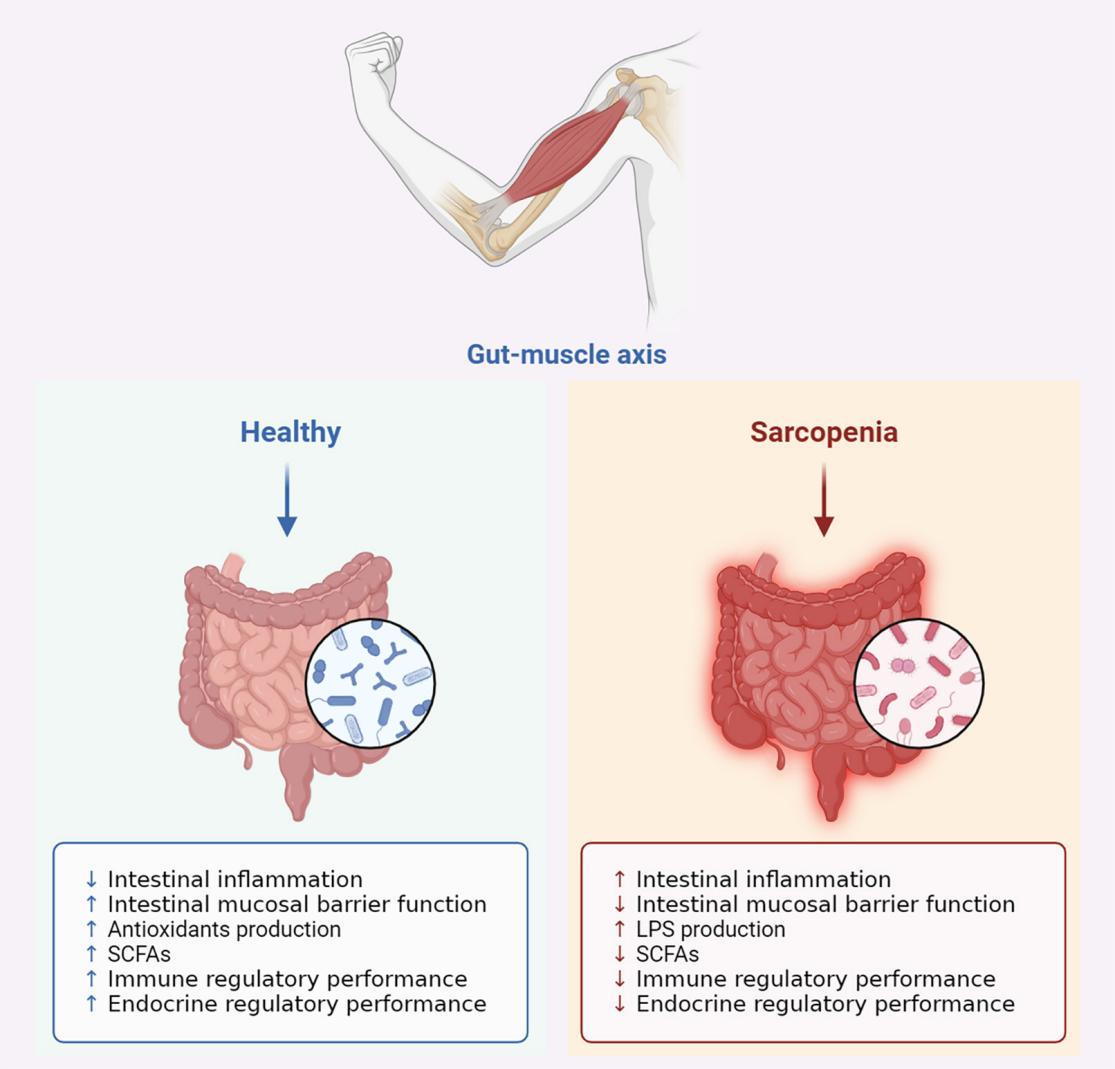
Schematic diagram of the regulatory mechanism of gut microbiota on sarcopenia based on gut–muscle axis. LPS, lipopolysaccharide; SCFAs, short‐chain fatty acids.

The pathogenesis of sarcopenia is complicated and involves multiple interactions, which is still in the research and exploration stage. Sarcopenia is mainly related to nutrition, exercise, hormones, inflammation, oxidative stress, immune disorders, neuropathy, hypoglycemia, multiple medications, mitochondrial abnormalities, lipid accumulation, insulin resistance, muscle satellite cells, glycosylation, pyrosis, apoptosis, autophagy, motor neuron degeneration, imbalance of muscle protein synthesis and catabolism, imbalance of cytokine dynamic regulation, genetic factors, and so on (Hashimoto et al. 2023; Kalinkovich and Livshits 2017; Larsson et al. 2019). In terms of gut–muscle axis, gut microbiota may participate in the occurrence of sarcopenia by modulating the butyrate and bile acid synthesis metabolism, inducing chronic low‐titer inflammatory reaction, promoting the insulin resistance, regulating muscle synthesis metabolism, affecting the host immune system, damaging tight junctions of intestinal epithelial cells, and enhancing intestinal permeability through various pathways (Nardone et al. 2021; Prokopidis et al. 2020). Moreover, partial microbial groups might play a crucial role in determining the muscle structure and function by producing metabolic mediators that influence host physiology after absorption through the intestinal mucosa (Bennett et al. 2023; Marques et al. 2023). Glycine betaine, tryptophan, bile acids, and butyric acid are the most promising biomarkers among putative mediators, and their discovery is considered a key research achievement in the field of sarcopenia worldwide (Daily and Park 2022; Prokopidis et al. 2021; Ticinesi et al. 2020). Collectively, the research progress on the pathogenesis of sarcopenia mainly includes the following aspects, and the schematic diagram of relevant mechanisms is exhibited in Figure 2.

### 4.1 Modulation of Inflammation and Intestinal Mucosal Barrier Function

Inflammatory reaction is regarded as one of significant factors in the occurrence and development of sarcopenia (Aluganti Narasimhulu and Singla 2021). Current studies have suggested that the level of inflammatory factors in the body is related to the mass or strength of skeletal muscle (Bano et al. 2017; Jimenez‐Gutierrez et al. 2022). With the aging of the body, the level of inflammatory markers in the circulatory system of the elderly is significantly enhanced, and it is directly related to the decline of muscle mass and strength in elderly (Pan et al. 2021). Therein, (1) tumor necrosis factor‐α (TNF‐α) can activate nuclear factor kappa‐B (NF‐κB) by inhibiting the Akt/mTOR pathway and by producing reactive oxygen species (ROS) via mitochondria (Vignaud et al. 2023); (2) inflammation can also accelerate the skeletal muscle catabolism (Nishikawa et al. 2021); (3) increased serum level of C‐reactive protein (CRP) is also associated with decreased protein synthesis and enhanced protein catabolism (Li et al. 2024); (4) interleukin‐6 (IL‐6) is involved in the regulation of muscle protein renewal and is considered as a catabolic cytokine in muscles (Xie et al. 2023).

Gut microbiota can produce immunogenic endotoxins, such as lipopolysaccharides (LPS), which are the components of the cell wall of Gram‐negative bacteria, and can induce the host inflammatory reactions (Chen et al. 2020; Eggelbusch et al. 2022; Zhang, Wang, et al. 2024). Experimental models of aging indicate that age‐related changes in the composition of gut microbiota increase the intestinal mucosal permeability. This phenomenon results in an enhancement of systemic absorption of bacterial products, including LPS, activating inflammatory responses and ultimately resulting in the elevated levels of proinflammatory cytokines in circulation (Liang et al. 2021). The number of LPS‐producing bacteria (such as *Enterobacteriaceae*) in the gut of elderly population increases, while the number of LPS‐inhibiting bacteria (such as *Bifidobacterium*) decreases (Pereira et al. 2022). Meanwhile, changes in intestinal epithelial cells increase the intestinal permeability and allow LPS to enter the circulation, and the interaction between LPS and macrophages results in the release of several inflammatory factors (Suzuki 2019). The correlation between the systemic inflammation, frailty, and sarcopenia has been previously verified (Ferrucci and Fabbri 2018; Mehta et al. 2021). From this perspective, systemic inflammation may represent one of the effectors of gut–muscle axis. Currently, it is believed that impaired intestinal barrier function can cause the displacement of gut microbiota and then lead to the inflammatory response of the body, which can promote the occurrence and development of sarcopenia (Maslennikov et al. 2023). In the context of gut–muscle axis, age‐related intestinal mucosal barrier dysfunction may play a central role in facilitating the entry of microbial products or microorganisms into the systemic circulation and aiding in the activation of inflammatory responses and inducing immune system dysfunction (Genton et al. 2021). Moreover, toll‐like receptors (TLRs) expressed in skeletal muscle tissue, such as TLR2, TLR4, and TLR9, can also recognize different pathogen‐related molecular patterns and ultimately activate the NF‐κB transforming factor in the skeletal muscle, leading to skeletal muscle atrophy (Khin et al. 2021; Verzola et al. 2017). Thus, it could be acknowledged that displaced intestinal bacteria may also mediate the occurrence and development of sarcopenia through TLRs/NF‐κB pathway.

### 4.2 Endocrine Modulation

With the aging of the body, the associated chronic inflammatory response can lead to the decrease of the levels of growth hormone (GH) and insulin‐like growth factor 1 (IGF‐1) and insulin resistance, induce protein synthesis resistance and the decline of skeletal muscle mass, and trigger the occurrence of sarcopenia (Vitale, Cesari, and Mari 2016; Ye et al. 2023). Currently, it has been experimentally verified that the protein production ability of aging muscle cells significantly decreases after being exposed to insulin, and microbial metabolite betaine can activate cytoplasmic calcium influx, extracellular signal‐regulated kinase (ERK) signal transduction, and IGF‐1 synthesis in human osteoblasts (Kim et al. 2021; Kuppusamy et al. 2019). In addition to this, the most extensively studied mediator for the impacts of gut microbiota on muscle function is SCFAs. SCFAs can enter the whole body through circulation and be absorbed by muscle cells (Lee et al. 2021). SCFAs can bind to the ligands of free fatty acid receptor 2 (FFAR2) and FFAR3 in skeletal muscle cells, and these receptors play a crucial role in regulating glucose uptake and metabolism, as well as promoting insulin sensitivity (El‐Deeb et al. 2023). The reduction of SCFAs may trigger insulin resistance, leading to an increase in intramuscular fatty acid deposition, and the subsequent decrease in muscle mass may further promote insulin resistance, form a vicious cycle, and promote the occurrence and development of weakness and sarcopenia (Lee, Shin, et al. 2023). Moreover, SCFAs can upregulate NAD‐dependent protein deacetylase sirtuin‐1 (SIRT‐1) receptor, which is a regulator of mitochondrial biosynthesis, and the expression of mitochondrial proteins is positively correlated with the average relative abundance of intestinal SCFA products in patients with inflammatory bowel disease (IBD), indicating a close link between mitochondrial function and gut microbiota (Erny et al. 2021). The mitochondrial proteins involved may determine the efficiency of energy production, the balance of redox reactions, and the regulation of inflammatory cascade activation. Meanwhile, researches have shown that systemic concentrations of acetate are associated with increased insulin resistance and obesity, and the presence of acetate may impair the skeletal muscle synthesis and have negative impacts on sarcopenia (Kumar et al. 2017). The biosynthesis of mitochondria in skeletal muscle cells may theoretically be regulated by secondary bile acids, which are synthesized by the primary bile acids via gut microbiota, and the occurrence of insulin resistance is also related to the imbalance of gut microbiota. Specifically, deoxycholic acid (DCA) is a free bile acid derived from the loss of an oxygen atom from cholic acid (CA). Both CA (primary bile acid) and DCA (secondary bile acid) are metabolites of bile acids and could activate TGR5. In this study, bile acids (CA and DCA) activate the ubiquitin–proteasome system, myonucleus apoptosis, and oxidative stress through a TGR5‐dependent mechanism, which induces mitochondrial dysfunction in skeletal muscles (Abrigo et al. 2022).

### 4.3 Disorders in the Synthesis and Absorption of Nutrients

The steady‐state imbalance of skeletal muscle protein synthesis and decomposition in elderly people is significant, and the decrease in protein intake and absorption leads to a decrease in muscle protein and fiber synthesis, which is lower than the breakdown of muscle protein, resulting in a vital decrease in muscle mass and strength (Miller et al. 2019; Wilkinson, Piasecki, and Atherton 2018). Gut microbiota has an efficient protein metabolism mechanism: Food and endogenous proteins are hydrolyzed into peptides and amino acids by proteases and peptidases produced by the host and bacteria, and the released amino acids are utilized by the host and microorganisms (Ticinesi, Lauretani, et al. 2019). Intestinal symbiotic bacteria play a crucial role in the separation, synthesis, and absorption of amino acids, such as alanine, aspartic acid, glutamic acid, glycine, and tryptophan (Grenier et al. 2023; Oh et al. 2021). Studies have shown that compared to pathogen‐free mice, germ‐free mice exhibit changes in the distribution of amino acids in the gastrointestinal tract, suggesting that gut microbiota may play an important role in amino acid balance and the health of the host (Sasabe et al. 2016). Gut microbiota can also utilize inorganic nitrogen, such as ammonium salts, to generate essential and nonessential amino acids for the human body. Partial subspecies of bacteria can even use the elemental nitrogen to synthesize organic nitrogen (Cosio and Powers 2023). Gut microbiota can synthesize partial amino acids, such as tryptophan, which is the basic substrate for skeletal muscle protein synthesis and metabolism (Jiang et al. 2022). Tryptophan can also stimulate the IGF1/p70s6k/mTor pathway in the skeletal muscle cells, thus promoting the expression of genes involved in myofibrillar synthesis (Dukes et al. 2015).

Additionally, the microorganisms in the human gut are also a significant pathway for supplying vitamins to the body. Normal bacterial communities in the human gut, such as *Bifidobacteria* and *Lactobacillus*, can synthesize various kinds of B‐group vitamins and vitamin K (Li, Zhou, and Gu 2016; Wong et al. 2018). The compounds, such as folate, vitamin B12, and tryptophan produced or modified by gut microbiota can enter the systemic circulation and ultimately affect skeletal muscle cells. It has been recognized that the deficiency of vitamin D and vitamin B12 may be a vital promoting factor for sarcopenia in elderly (Borda et al. 2024). The normal gut microbiota might improve skeletal muscle synthesis and metabolism by producing folate and vitamin B12 and prevent the oxidative stress and endothelial damage caused by hyperhomocysteinemia, resulting in decreased skeletal muscle function (Nasimi et al. 2023). In addition to vitamin B12, gut microbiota could also affect the absorption of vitamin D. Previous studies have revealed that the group with a high proportion of *Prevotella* in the gut microbiota has the best absorption of vitamin D, while *Bifidobacterium* are negatively correlated with 25 hydroxyvitamin D (Boughanem et al. 2023). Meanwhile, studies have also indicated that compared to rats with normal muscle mass, rats with age‐related skeletal muscle wasting exhibit different compositions of gut microbiota, and the microbiota of rats with muscle wasting also exhibits different functions, with reduced gene expression involved in carbohydrate, protein, lipid digestion, and vitamin biosynthesis (Nikkhah et al. 2023; Ticinesi et al. 2023). Collectively, reduced macronutrients and vitamin bioavailability associated with these age‐related functional changes may play a key pathophysiological role in determining the muscle mass loss.

## 5 Potential Methods to Alleviate Sarcopenia by Modulating Gut Microbiota

**FIGURE 3:**
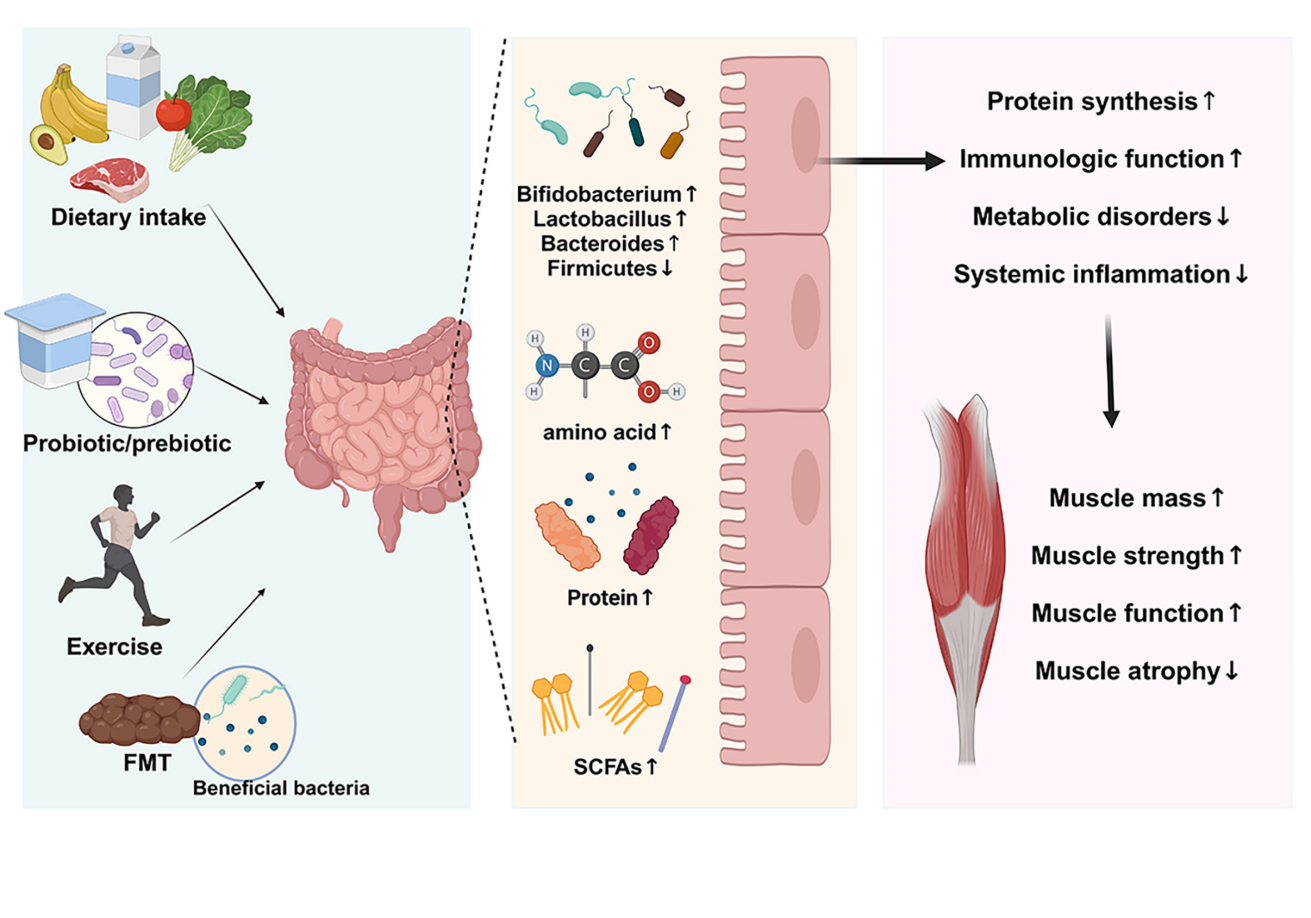
Potential methods to alleviate sarcopenia by modulating gut microbiota.

Currently, interventions for the elderly with sarcopenia are mainly nutritional and exercise interventions, including the dietary intake, probiotic/prebiotic supplementation, fecal microbiota transplantation (FMT), exercise, and so on (Casati et al. 2019; Liao et al. 2020; Lobo et al. 2020). To date, there are few researches evaluating the impacts of changes in gut microbiota on the skeletal muscle quality and function, and most of these are focused on animal models, lacking high‐quality population‐based cohort studies (Pellanda, Ghosh, and O'Toole 2021). Collectively, the following elaborates on the potential methods to alleviate sarcopenia by modulating gut microbiota, as shown in Figure 3.

### 5.1 Dietary Intake

Currently, it is widely recognized that proteins, amino acids, and dietary fiber play a crucial role in maintaining muscle stability and homeostasis of gut microbiota (Zhang, Cao, Li, Sheng, et al. 2023). Researches have indicated that supplementing 1.2–1.5 g/kg of protein daily can enhance muscle mass and strength, thus delaying the occurrence and development of sarcopenia (Motiani et al. 2020). Protein, as an essential nutrient for the human body, can also participate in the composition of gut microbiota. Sergeev et al. (2020) revealed that a high‐protein diet can significantly enhance the abundance of bacterial colonies, such as *Bifidobacterium* and *Lactobacillus*. Meanwhile, essential amino acids could stimulate protein synthesis in patients with sarcopenia and contribute to the body to overcome protein resistance, enhance muscle mass, and improve body performance, and it is also recommended to choose the amino acid supplements rich in levamic acid (Aussieker et al. 2023). In addition, dietary fiber can not only affect muscle function but also influence the composition and metabolism of gut microbiota (Zhang, Hu, et al. 2024). Researches have suggested that a high dietary fiber could promote the growth of beneficial bacteria and enhance the immune function of body, which is conducive to maintaining healthy intestinal microecology (Zhang, Zhou, et al. 2024). Scholars have also found that a high dietary fiber diet can weaken the adverse effects of metabolic disorders on the muscle mass regulation by enhancing the richness of gut microbiota and circulating free fatty acid concentrations (He et al. 2024; Hevia‐Larraín et al. 2021). Moreover, Kahleova et al. (2020) recruited 168 participants for a 16‐week experiment on the effects of a high‐fat diet and a low‐fat vegan diet on gut microbiota, and the results indicated that the participants on a low‐fat vegan diet had an enhancement in the number of *Bacteroides* and a decrease in the level of *Firmicutes*. More importantly, the balanced ratio of the above two bacterial colonies can also maintain the homeostasis of gut microbiota and muscle mass. A systematic review indicated that a high‐fat diet not only induces a decrease in the number of gut microbiota but also mediates the degradation of muscle branched chain amino acids, creatine, and other nutrients, causing muscle mass and functional impairment by influencing the gut–muscle axis (Liu et al. 2021). Collectively, dietary nutrition intervention is of crucial significance for the population with sarcopenia, and researchers should actively pay attention to the nutritional status of sarcopenia populations and deeply explore the regulatory effects of gut microbiota on dietary intake for sarcopenia.

### 5.2 Probiotic/Prebiotic Supplementation

Nutritional supplements, such as probiotics and prebiotics, may be involved in processes such as protein synthesis and lipid metabolism, effectively enhancing muscle mass and body function (Kang et al. 2024; Zhang, Li, et al. 2024). Therein, probiotics include several genera, among which *Bifidobacterium* and *Lactobacillus*, as the most common probiotics, play a role in maintaining homeostasis in the body. For example, the supplementation of *Bifidobacterium* can regulate Gram‐negative bacteria to maintain normal numbers and reduce the absorption of toxins (de Marco Castro, Murphy, and Roche 2021). The supplementation of *Lactobacillus* can also increase the absorption of proteins, calcium, and other substances, while also reducing the intestinal pH and promoting intestinal motility and absorption (Taslim et al. 2023). The intake of prebiotics can improve gastrointestinal function and muscle mass by regulating SCFAs. A previous study revealed that the probiotic supplements composed of inulin and oligofructose may have positive effects on the healthy microbiota, while also enhancing hand strength and endurance in patients with sarcopenia (Daily and Park 2022). Currently, there are few researches evaluating the effects of probiotic/prebiotic supplementation on muscle mass and function, and most of these studies focus on animal models. In recent years, it has been found that the grip strength of mice after consuming *Lactobacillus* is significantly higher than that of the control group, which is also positively correlated with the dose intake (Baek et al. 2023). Bindels et al. (2012) investigated that the supplementation of *Lactobacillus* could improve systemic inflammation and muscle atrophy in BAF mice, which can improve the expression of muscle mass loss and muscle atrophy markers in tibia and fibula of mice. Collectively, probiotic/prebiotic supplementation is a promising approach for the prevention and treatment of sarcopenia, while it is still in the early stages of research, and further, larger‐scale population studies are still needed to validate it in the future.

### 5.3 Exercise

Exercise, as the most effective nonpharmacological intervention for sarcopenia, has a positive effect on maintaining muscle mass and maintaining stable neuromuscular junction function. Currently, the recommended exercise intervention methods mainly include resistance exercise, group comprehensive training, and whole‐body vibration training (Hurst et al. 2022; Shen et al. 2023). Zhu et al. (2019) selected 113 patients with sarcopenia and conducted 12 weeks of resistance training under guidance, including two sessions/week of 90‐min group training and one session/week of home training, and the results suggested that the resistance exercise significantly improved the lower limb muscle strength of patients. Meanwhile, exercise also has positive effects on the gut microbiota. In a previous study, compared with the rat in the sedentary group, physical activity group not only significantly increased cardiovascular endurance but also optimized the structure of gut microbiota and inhibited harmful bacteria (Morifuji et al. 2021). Marttinen et al. (2020) also pointed out that the abundance of SCFAs may increase in the individuals who regularly exercise. Consistent with this, a randomized controlled trial showed that exercise can reduce the level of inflammation in the body and regulate gut microbiota, mainly manifested in a decrease in the ratio of *Firmicutes* and *Bacteroidetes* (Moreno‐Pérez et al. 2018). Therein, *Bacteroidetes* can weaken the release of inflammatory products from gut microbiota, and *Bacteroidetes* are also the main source of SCFAs (Hu et al. 2020). Hence, it can be speculated that the alterations of gut microbiota caused by exercise may be related to SCFAs. Overall, exercise intervention has a positive effect on the condition and intestinal microbiome homeostasis of patients with sarcopenia. However, there is currently no specific standards on the time, intensity, and mode of exercise, and there is a lack of relevant guidelines, which is the direction of attention and efforts in the future.

### 5.4 FMT

Recently, researches have also shown that the application of FMT can regulate and rebuild the composition of gut microbiota and its related metabolites and contribute to reduce adverse reactions, such as malnutrition and muscle loss caused by sarcopenia (Kim et al. 2022; Zhang, Cheng, and Hu 2022). With the assistance of FMT, gut microbiota and its related metabolites can reverse the quality of skeletal muscle, improve muscle function, and alleviate or prevent sarcopenia (Xiao et al. 2024). Fielding et al. (2019) also revealed that the muscle strength of germ‐free mice transplanted with fecal bacteria of the elderly with different body functions is significantly improved compared with the germ‐free mice transplanted with the fecal bacteria of the elderly with low function. Moreover, researchers have transferred gut microbiota from obese model mice to sarcopenia model mice by means of FMT and suggested that the metabolic and fiber characteristics of skeletal muscles in the mice have undergone alterations like those of donors, which indicates that FMT has a certain regulatory effect on the function of skeletal muscles, and FMT is expected to become a clinical method for alleviating or preventing sarcopenia (Lapauw et al. 2023).

## 6 Conclusions and Perspectives

Currently, although there is an enhancing number of researches demonstrating the correlation between gut microbiota and sarcopenia, there is still relatively little research on the etiology and intervention between gut–muscle axis. Specifically, the mechanism of action of the gut–muscle axis is not yet clear, and there is a lack of research evidence to support how factors, such as aging and disease, affect sarcopenia and gut microbiota. Regarding this, further investigations are still needed in the future to determine whether intervention on the gut–muscle axis could delay the muscle atrophy, improve the functional impairment, and enhance the richness of gut microbiota. Partial key gut microbiota may affect the host physiology by producing metabolites, such as glycine, tryptophan, bile acids, and SCFAs, which are absorbed by intestinal mucosa and play related roles in muscle structure and function. These molecules in stool, as well as the bacteria that produce it, may be the biomarkers of sarcopenia, and its detection may become a focus of research in the field of sarcopenia. Moreover, there are relatively little researches on the roles and mechanisms of gut microbiota intervention in sarcopenia, and researches on probiotics or their effects on muscle intervention mainly focus on animal models; whether the results can be translated into humans is still uncertain. Thus, future studies should focus more in‐depth on gut–muscle axis and its clinical‐related contents. Ultimately, current studies on the relationship between gut microbiota and sarcopenia are mainly limited to literature reviews or small sample studies. It is recommended to select patients from different regions, ages, populations, and dietary habits for large‐scale clinical trials, thus conducting the relevant evidence‐based studies on the measures, such as dietary intake, probiotic/prebiotic supplementation, exercise interventions, and FMT, to provide more instructive research programs in the future.

## Ethics Statement

The authors have nothing to report.

## Consent

The authors have nothing to report.

## Conflicts of Interest

The authors declare no conflicts of interest.

## Data Availability

The authors have nothing to report.
